# Gene-Related Response of Basal Cell Carcinoma to Biologic Treatment with Vismodegib

**DOI:** 10.1038/s41598-020-58117-0

**Published:** 2020-01-27

**Authors:** Amir Sternfeld, Shirel Rosenwasser-Weiss, Gur Ben-Yehuda, Hila Kreizman Shefer, Moran Friedman-Gohas, Iftach Yassur, Gil Tauber, Jacob Bejar, Asaf Olshinka, Yoav Vardizer, Dean Ad El, Nitza Goldenberg-Cohen

**Affiliations:** 1 Department of Ophthalmology, Rabin Medical Center – Beilinson Hospital, Petach Tikva, Israel; 2 The Krieger Eye Research Laboratory, Felsenstein Medical Research Center, Petach Tikva, Israel; 3 Sackler Faculty of Medicine, Tel Aviv University, Tel Aviv, Israel; 4 Department of Plastic Surgery, Rabin Medical Center – Beilinson Hospital, Petach Tikva, Israel; 5 Department of Pathology, Bnai Zion Medical Center, Haifa, Israel; 6 Department of Dermatology, Rabin Medical Center – Beilinson Hospital, Petach Tikva, Israel; 7 Department of Ophthalmology, Bnai Zion Medical Center, Haifa, Israel; 8 The Ruth and Bruce Rappaport Faculty of Medicine, Technion-Israel Institute of Technology, Haifa, Israel

**Keywords:** Cancer genetics, Cancer genomics, Basal cell carcinoma

## Abstract

We aimed to characterise the response of locally advanced basal cell carcinoma (BCC) to systemic treatment with Vismodegib, a Hedgehog pathway inhibitor, by changes in the expression levels of Hedgehog pathway genes. Data were collected prospectively on 12 patients treated systemically for locally advanced BCC. Biopsy samples taken on admission and after treatment cessation were analysed pathologically and with the NanoString nCounter system to quantify the expression of 40 Hedgehog signaling pathway genes. Findings were compared before and after treatment, between complete and partial responders, and with localised BCC samples from 22 patients. Sixteen Hedgehog pathway genes changed significantly from before to after treatment. *GAS1* was the only gene with a significantly different expression at baseline between complete responders (6 patients) and partial responders (4 patients) to Vismodegib (*P* = 0.014). GAS, *GLIS2* and *PRKACG*1 showed different expression before treatment between the locally advanced and localised BCCs. The baseline expression level of *GAS1* appears to be predictive of the response of locally advanced BCC to systemic Vismodegib treatment. A change in expression of many Hedgehog pathway genes, albeit expected by the known activity of Vismodegib, may nevertheless serve as an indicator of the response potential of the tumour.

## Introduction

Basal cell carcinoma (BCC) is the most common cancer in the world, with a lifetime risk of 20% and an incidence that has grown continuously over recent decades^1,2^. BCC accounts for 80% of all nonmelanoma skin cancers. It occurs with greatest frequency in Caucasian and elderly populations^1,2^. The tumours are generally slow-growing, rarely fatal (mortality rate, less than 0.1%), and seldom metastasise (up to 0.5% of all cases)^1,2^. For localised BCCs, surgical treatment has a 5-year cure rate of more than 95%^3,4^. However, for the minority of BCCs that are locally advanced (laBCC) or metastatic (metBCC), conventional treatment methods are inadequate, and systemic therapies are considered the appropriate approach^2^.

The Hedgehog (Hh) signal transduction pathway is one of the key regulators of cell proliferation and differentiation during embryogenesis and plays a major role in ensuring proper embryonic development^5,6^. In adults, the Hh pathway is normally inactive except in stem and skin cells and hair follicles. However, studies have shown that aberrant uncontrolled activation of the Hh pathway is maintained in 95% of sporadic BCCs in addition to several other malignancies^7^.

**Figure 1:**
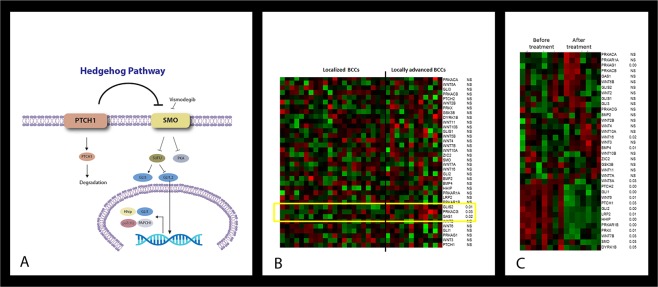
(**A**) Hedgehog (Hh) pathway. (**B**) Heatmap demonstrating differences in Hh pathway gene expression between locally advanced BCC (laBCC) and localised BCC tumours. (**C**) Heatmap demonstrating differences between pre- and post- treatment Hh pathway gene expression in the laBCC group.

The Hh pathway is activated when the Hh ligand binds to and inhibits the Patched 1 (PTCH1) receptor, an inhibitor of the G-protein-coupled smoothed (SMO) transmembrane receptor. As a result, the activated SMO then inhibits the negative regulator Suppressor of Fused protein which binds glioma associated (GLI) transcription factors in the cytoplasm. The GLI transcription factors are then left free to enter the cell nucleus and promote cell division and tumourigenesis (Fig. 1A)^5,6,8^. Approximately 90% of sporadic BCCs demonstrate mutations in the *PTCH1* gene, often associated with loss of heterozygosity; most of the remaining 10% show a gain-of-function *SMO* gene mutation. Both these mutations cause uncontrolled proliferation of skin basal cells^9,10^.

Vismodegib (Erivedge^®^ Capsule, Genentech Inc., South San Francisco, CA, USA) is a first-generation synthetic small-molecule SMO receptor inhibitor. In 2012, the ERIVANCE study reported a 30% response rate to Vismodegib for metBCC and a 43% response rate for laBCC^11^. Prompted by these findings, the United States Food and Drug Administration approved the use of oral Vismodegib for the systemic biologic treatment of adults with laBCC or metBCC. The aim of the present study was to investigate the effect of Vismodegib on the expression levels of Hh pathway genes in laBCC and to search for potential predictors of tumour response.

## Results

Twelve patients with laBCC and 22 patients with localised BCC were recruited for the study. The patient characteristics and clinical data are summarised in Table 1. Four patients in the laBCC group were excluded from the post-treatment analysis for the following reasons: receipt of a reduced dose of Vismodegib (n = 1); treatment with radiation prior to biopsy study (n = 1); tissue quality was too poor for evaluation (n = 1); or the biopsy sample was taken 6 months after cessation of treatment, when BCC recurred (n = 1).

**Table 1:** Demographics and clinical characteristics of the patients at baseline.

Characteristics	Locally advanced BCC (N = 12)	Localised BCC (N = 22)
Age (y), mean ± SD (range)	79.1 ± 8.1 (60–88)	71.5 ± 14.4 (38–90)
Sex		
Male	6 (50%)	16 (72.7%)
Female	6 (50%)	6 (27.3%)
Tumor location		
Head and neck	10 (83%)	14 (63.6%)
Trunk and limbs	2 (17%)	8 (36.4%)
Contraindication for surgery		N/A
Inoperable tumor	3 (33.3%)	
Substantial morbidity or deformity anticipated	6 (66.7%)	
Response to treatment		N/A
Complete response	6 (55.5%)	
Partial response	4 (36.4%)	
No response	1 (9.1%)	

Data are presented as n(%) unless otherwise stated.

BCC, basal cell carcinoma.

Comparison of the laBCC tumours before systemic treatment with localised BCC tumours yielded a significant differential expression of three genes of the Hh pathway: *GAS1*, GLI similar 2 (*GLIS2)*, and protein kinase CAMP-activated catalytic subunit gamma (*PRKACG)* (*P* < 0.05; Table 2). A heatmap demonstrating the similarity of the groups in the expression of all Hh pathway genes except these three is shown in Fig. 1B.

**Table 2:** Hedgehog pathway genes with a differential expression in localized and locally advanced BCC tumors before treatment.

Gene	Locally advanced BCC	Localised BCC	*P* value
GAS1	2440.19 ± 1200.14	1694.39 ± 582.98	0.019
GLIS2	72.66 ± 38.73	39.51 ± 27.02	0.006
PRAKCG1	3.8 ± 3.98	1.76 ± 1.21	0.031

BCC, basal cell carcinoma.

On comparison of the levels of expression of the Hh pathway genes before and after Vismodegib treatment in the laBCC group, a significant qualitative change was found in 16 genes (*P* < 0.05), as shown by the heatmap in Fig. 1C. They included several pivotal genes from the *GLI, PTCH*, and *WNT* families and *SMO* (Table 3). The patients (n = 11, with the exclusion of the patient given a reduced dose) were then divided by the endpoint of treatment according to the RECIST guidelines. Six patients had a complete response, 4 patients had a partial response, and one patient failed to respond. The latter patient was excluded from the analysis for statistical purposes. Molecular analysis revealed that the mean level of expression of *GAS1* was significantly lower in the patients with a complete response compared to the patients with a partial response: 1874.83 ± 905.26 vs. 3901 ± 420.05, respectively (*P* = 0.014). This was the only Hh pathway gene showing a significantly different expression between these subgroups. There was no difference between the subgroups in the expression of *GLI1* (1958.73 ± 1052.86 vs. 1753.17 ± 1539.18, respectively, *P* = 0.842).

**Table 3:** Hedgehog pathway genes showing a significant change in expression (*P* < 0.05) from before to after Vismodegib treatment of locally advanced BCC.

Gene	Before treatment	After treatment	*P* value
BMP4	251.09 ± 126.35	414.41 ± 98.86	0.012
GLI1	1962.4 ± 1387.05	48.31 ± 46.38	0.001
GLI2	5671.2 ± 4064.22	588.68 ± 243.98	0.003
HHIP	511.82 ± 341.94	2.76 ± 1.08	0.000
LRP2	416.64 ± 383.36	2.76 ± 1.085	0.008
PRKAG1	798.35 ± 185.21	1241.32 ± 234.03	0.000
PRKAR1B	1870.50 ± 1125.40	313.72 ± 121.02	0.001
PRKX	1060.93 ± 561.7	401.26 ± 159.29	0.006
PTCH1	4047.11 ± 4424.32	176 ± 136.69	0.026
PTCH2	12086.3 ± 6592.69	287.09 ± 409.22	0.000
SMO	1205.88 ± 494.02	698.32 ± 331.9	0.03
WNT16	272.98 ± 247.72	949.41 ± 680.75	0.019
WNT5A	4438.66 ± 2233.29	2313.95 ± 1270.9	0.034
WNT6	239.06 ± 217.6	16.84 ± 14.21	0.012
WNT7B	1742.1 ± 1849.32	171.24 ± 135.07	0.031
DYRK1B	946.535 ± 274.06	715.65 ± 118.35	0.046

BCC, basal cell carcinoma.

Immunostaining to GAS1 on 4 localized BCC samples was compared to one normal skin sample in an attempt to show the feasibility of the staining. However, the BCC samples showed decreased staining compared to the normal skin, which precluded us from performing further staining on the complete and partial responders due so small amount of tissue remaining.

The localised BCCs were also compared by site: head and neck (n = 13), where sun exposure over time is substantial, and trunk (n = 9), where sun exposure is limited. A significant difference in three Hh pathway genes was noted between these subgroups: *GLI3* (2207.11 ± 693.83 vs. 2853.74 ± 739.30, respectively, *P* = 0.049), protein kinase CAMP-dependent type 1 regulatory subunit beta (*PRKAR1B;* 2642.98 ± 1476.28 vs. 1444.93 ± 743.84, respectively *P* = 0.037), and wingless/integrated 2 (*WNT2;* 173.54 ± 141.20 vs. 39.75 ± 31.82, respectively *P* = 0.012).

## Discussion

This study presents a comprehensive analysis of the response of Hh signalling pathway genes to treatment with Vismodegib in patients with laBCC. We found that before treatment, laBCCs were characterised by a significantly higher level of expression of three Hh pathway genes compared to localised BCCs. After systemic treatment of laBCC with Vismodegib, the expression of 16 Hh pathway genes changed from baseline. Division of the laBCC group by partial or complete response to treatment showed that a single gene, *GAS1*, was predictive of the tumour response.

NanoString nCounter analysis successfully revealed a significant decrease in the expression of many of the Hh pathway genes located downstream to the Vismodegib-inhibited SMO receptor. The ability of the NanoString nCounter system to quantify a large amount of mRNA simultaneously makes it a potential tool for determining tumour response to treatment and for identifying genes with consistent overexpression in resistant tumours that might be targeted for second-line therapy.

Further analysis to identify which of the genes could have predictive value in terms of tumour response to systemic treatment yielded only one, *GAS1*, which had a significantly higher level of expression in tumours with a partial response than in those with a complete response. The GAS1 protein modulates Hh pathway signalling together with two other cell-surface proteins, CAM-related/downregulated by oncogenes (CDO) and Brother of CAM-related/downregulated by oncogenes. GAS1 is the only one of the three that exists solely in vertebrates^12,13^. These 3 proteins function as coreceptors for the Hh ligand. They interact with the PTCH1 protein on the cell surface, increase its affinity for the ligand and thus enhance the Hh pathway^14,15^. Therefore, they potentially contribute to the excessive activation of the pathway, and several authors have already suggested their possible role in tumourigenesis^15,16^.

As Vismodegib inhibits the Hh pathway downstream to the activity of *GAS1*, its effect would not be expected to be influenced by the higher expression of *GAS1*. It is possible that the overexpression of *GAS1* potentiates the pathway enough to overcome Vismodegib inhibition by releasing more SMO from PTCH1. Another possible explanation is that *GAS1* has some effect, even if relatively mild, on SMO as well.

Patched 2 (PTCH2) is a second patched member, structurally similar to PTCH1, whose role as a tumour suppressor and response to Hh ligand have yet to be entirely understood. Several groups provided proof for at least some activity as a tumor suppressor and response to the Hh ligand, similarly to PTCH1^17,18^. It is plausible that GAS1 also interacts with PTCH2 as a coreceptor. Thus, when GAS1 is overexpressed, PTCH2 has increased affinity to Hh ligand that inhibits its activity as a SMO inhibitor. This additional activation of SMO receptors (together with the mutated PTCH1 or SMO genes) could improve the tumour’s ability to resist some of the inhibition caused by Vismodegib, explaining the high GAS1 levels in the partially responsive laBCCs.

Interestingly, *GAS1* was recently found to play a protective role against tumourigenesis and metastasis in colon and gastric cancers^16,19,20^. Its ability to suppress the tumour was attributed to its participation in the negative regulation of aerobic glycolysis, an essential requirement for tumour growth and spread^19–21^. In our study, the higher pretreatment *GAS1* levels in the partially responding laBCCs may suggest that these tumours were more aggressive and therefore able to spread even with these *GAS1* levels, making them relatively resistant to the systemic treatment.

*GAS1* was one of only three Hh pathway genes that were differentially expressed in laBCCs and localised BCCs. The overexpression of *GAS1* in laBCC may correspond to its higher level of expression in the partially treatment-responsive than in the completely responsive tumours. The importance of the other two genes, *GLIS2* and *PRAKCG*, in this context has yet to be explored. The GLIS2 protein is a member of a subfamily of transcription factors that have a great impact on different physiological processes, including cancer. In contrast to GLIS1 and GLIS3, which are considered to be solely transcriptional activators, GLIS2 is also a repressor. However, there are no available data on its specific role in the Hh pathway or in BCC^22^. PRAKCG is part of the protein kinase C family which regulates cell differentiation and proliferation and inhibits apoptosis. Specifically, it was recently associated with the pathogenesis of several diseases and tumours, including colon and breast cancer. Its possible role in the Hh pathway or in BCC has not been investigated^23,24^.

Unfortunately, our attempt to use immunohistochemical staining to show overexpression of GAS1 in the partially vs. complete responsive laBCC was unsuccessful. We attempted to show the feasibility of the staining by comparing the expression of GAS1 in localised BCC vs. normal skin prior to using the remaining laBCC tissue for this staining. However, the results showed decreased staining in the BCC samples comparing to normal skin. This discrepancy between the immunohistochemical staining and the mRNA overexpression could be attributed to the fact that not all mRNA are translated to proteins. We suggest future studies using *in situ* hybridization staining to localise and determined the actual mRNA levels in the cells.

*GLI1* expression is considered the most important indicator of the Hh pathway activity. There are three GLI transcription factors (GLI1-3): GLI1 is a full-length transcriptional activator, and GLI2 and GLI3 regulate the activity of GLI1^5,8^. Atwood *et al*.^7^ recently compared the levels of GLI1 mRNA between normal skin, Vismodegib-sensitive and Vismodegib-resistant BCCs. Levels were slightly elevated in the sensitive tumours and high in the resistant tumours, suggesting the presence of genetic alterations that maintained the elevated output of the Hh pathway genes despite treatment. In the present study, there was no difference in the pretreatment levels of *GLI1* between the tumours with a partial or complete final response or between localised BCCs and laBCCs. The high pretreatment levels may be explained by an upregulation of the Hh pathway in all BCCs. We speculate that during treatment, there is a drop in levels of GLI1, as an end product of the Hh pathway, unless the tumour successfully maintains excessive activity of the Hh pathway via bypass pathways or adjunctive mutations that the treatment cannot overcome.

A previous study investigated gene levels in BCCs compared to body surface areas exposed to sun^25^ and found that the BCCs had an overexpression of *WNT2* and *PRKAR1B* together with an underexpression of *GLI3*. The Wnt pathway, similar to the Hh pathway, plays a pivotal role in embryonic development^25^, and overexpression of *WNT* has been identified in several types of cancers, causing proliferation, migration, and invasion of the tumour cells. The affected cancers included human melanoma and squamous cell carcinoma of the head and neck, both of which, like BCC, are related to sun exposure^26–29^. Further research should be directed at determining whether exposure to the sun is a direct cause of damage to these genes.

The main limitation of this study is the small sample size, a direct result of the relatively infrequent use of systemic treatment for BCCs. A large multicenter study is warranted. *In situ* hybridization staining should be used to confirm the findings and localizing the mRNA in future studies.

In summary, this study is the first to identify *GAS1* baseline levels as a possible marker for the response of laBCCs to Vismodegib. This could lay the ground for future research of the role of *GAS1* in the Hh pathway in general and in the resistance of the tumour to biologic treatment with Vismodegib. *GAS1* might also serve as a target for future treatments.

## Methods

### Study design and patients

A prospective study was conducted at a tertiary medical center that serves as a national center for the systemic treatment of laBCC and metBCC. The study adhered to the tenets of the Declaration of Helsinki and was approved by the local institutional review board of Rabin Medical Center. The cohort included all patients with laBCC who were treated with oral Vismodegib between January 2015 and September 2016 and signed an informed consent to participate in the study. Exclusion criteria were previous tumour treatments with other modalities.

The patients were evaluated clinically by a dermatologist (G.T.) and a plastic surgeon (G.B.Y., D.A.) before and after cessation of treatment. The response to treatment was determined according to the RECIST guidelines^30^. Biopsy samples were taken for histological study and to molecularly quantify the expression of the different Hh pathway genes at both time points. The findings were compared from before to after treatment, between complete and partial responders, by tumour site and with samples of localised BCC taken from patients prior to tumour excision.

### Molecular analysis procedure

Biopsy samples were fixed in formalin and embedded in paraffin, and 8–10 µl of mRNA were extracted at a concentration of 100–150 ng/µl using the RNeasy FFPE Kit (QIAGEN, Germany). The molecular analysis was performed by an external company using the NanoString nCounter system (Agentek Ltd., Israel) which directly quantifies gene expression with high precision and sensitivity. The system is capable of counting hundreds of unique mRNA transcripts in a single reaction without the need for reverse transcription or amplification^31^. For the present study, the expression levels of 40 genes in the Hh pathway were analysed in each sample from the patients and controls.

### Tissue processing for immunohistochemistry

Four samples of BCC and one sample of normal skin were fixed in 4% paraformaldehyde, processed routinely and embedded in paraffin. Formalin fixed paraffin-embedded sample sections (3 μm) were mounted on superfrost slides. Hematoxylin and eosin staining was used for histological evaluation under light microscope. Sequential sections were used for GAS-1 staining.

### GAS1 immunohistochemistry

The immunostains were performed on an automated stainer (Benchmark Ultra; Ventana Systems, Phoenix, AZ). Primary antibody incubation time was 28 minutes after antigen retrieval in a Tris based buffer (20 minutes at 95–100 °C). Polyclonal rabbit anti-human GAS1 antibody (17903–1-AP, Proteintech) (diluted 1:50) was used. The detection reaction used the ultraVIEW DAB detection kit (760–500 Ventana Systems, Phoenix, AZ), according to manufacturer-recommended protocol. Hematoxylin counterstain was used for color development.

### Statistical analysis

Data for continuous variables are presented as mean and standard deviation and for nominal parameters as number. Unpaired Student’s t-test was used to compare gene expression levels in laBCC samples before and after treatment, between complete and partial response, with the localised BCC and between sites of localised BCC. A *P* value of < 0.05 was considered significant. All statistical analyses were performed using SPSS for Windows (SPSS, Inc. Chicago, IL, USA).

## Data Availability

All data generated or analysed during this study are included in this published article.
